# Cu_2_O, ZnO, and Ag/ Cu_2_O nanoparticles synthesized by biogenic and chemical route and their effect on *Pseudomonas aeruginosa* and *Candida albicans*

**DOI:** 10.1038/s41598-023-47917-9

**Published:** 2023-12-06

**Authors:** J. Rojas-Jaimes, David Asmat-Campos

**Affiliations:** 1 Faculty of Health Sciences, Universidad Privada del Norte, Av. El Sol 461, San Juan de Lurigancho, Lima, 15434 Peru; 2 Department of Research, Innovation & Social Responsibility, Universidad Privada del Norte, Trujillo, Peru; 3 Applied Sciences and New Technologies Research Group, Universidad Privada del Norte, Trujillo, Peru

**Keywords:** Biotechnology, Microbiology, Materials science

## Abstract

*Pseudomonas aeruginosa* and *Candida albicans* are two important pathogens in public health due to the infections they cause in immunocompromised patients and with hospital stay, increasing morbimortality rates. Three groups of Cu_2_O, ZnO, and Ag/Cu_2_O nanoparticles were synthesized and characterized physicochemically and confronted to *P. aeruginosa* and *C. albicans* to determine their antibacterial effect. Statistical analyses were performed using Analysis of Variance (ANOVA) (p < 0.001). The structures of Cu_2_O, ZnO, and Ag/Cu_2_O nanoparticles were spherical, sized 6 nm, 10 nm, and 50 nm for Ag, Cu_2_, and Zn metals, respectively. Furthermore, a 100% antibacterial and antifungal effect against *Pseudomonas aeruginosa* and *Candida albicans* was observed for Cu_2_O, ZnO, and Ag/Cu_2_O nanoparticles respectively. It is concluded from these findings that the nanoparticles synthesized by biogenic and chemical route had a good size between 6 and 50 nm and that Cu_2_O, ZnO, and Ag/Cu_2_O nanoparticles presented an excellent antibacterial (100% growth inhibition) effect against *P. aeruginosa* and *C. albicans* (p < 0.001) compared to the control.

## Introduction

In recent years, much emphasis has been placed on research on nanoparticles (NPs), in addition to the importance of the environment, eco-friendly methods are being investigated in the use of reducers for the synthesis of nanoparticles, these being more economical and generating less pollution. The ecological methods include the use of extracts of leaves, stems and fruits^1–3^. Reducing substances in plant extracts are the ones that react in the reduction process and the formation of NPs^4,5^. Plant extracts such as *Eucalyptus camaldulensis, Azadirachta indica, Murraya koenigii, Avicennia marina, Rosa rubiginosa* and *Datura stramonium*, have been used in the reduction to create CuO NPs, characterizing the sizes of these NPs between 29 and 48 nm^6^. The synthesis of NPs is dependent on the pH, temperature and concentration of the plant extracts. In addition, it has been found that at alkaline pH and at a temperature of 60 °C the NPs obtained are smaller^7^.

Recent studies have determined that CuO nanoparticles have remarkable antibacterial and antifungal effects^8–10^. This supports the use of copper nanoparticles against *P. aeuriginosa* and *C. albicans,* which are agents of importance in public health for causing nosocomial infections. The extracts of *Eucalyptus globulus* and *Mentha piperita* have been used with good results against *Colletotrichum capsici*, a fungus of agro-industrial importance^11^, In the case of the use of *Citrus sinensis* for the synthesis of CuO Nps, it showed a noticeable effect against *Escherichia coli* and *Staphylococcus aureus* without showing a cytotoxic effect on mouse fibroblasts^12^.

The search for antibacterial and antifungal substances have led to the development of the synthesis of NPs such as silver impregnated in wool and cotton^13–15^. In an investigation on the synthesis of silver and copper NPs in impregnated cotton, its effect against *Klebsiella pneumoniae, Candida albicans* resistant to antibiotics was confirmed^16^. The effect of silver and the hybrid of copper and silver impregnated in a substrate could serve as an antimicrobial in hospital bandages although the price of a hybrid and silver nanoparticle could be more expensive than copper nanoparticles.

The study determined the antimicrobial effect of Cu_2_O, ZnO, and hybrid (Ag/Cu_2_O) nanoparticles synthesized by biogenic and chemical route and their effect on *Pseudomonas aeruginosa* and *Candida albicans.*

## Methodology

### Green synthesis of silver nanoparticles (Ag NPs)

A precursor solution of silver nitrate ACS (CAS no. 7761-88-8) at 0.01 mM was used for synthesizing nanomaterial, and the mixture was kept under magnetic stirring until brought to 60 °C. Then, a burette was loaded with 25 mL of 96% alcoholic extract of *Eucalyptus globulus* (previously filtered) and was added dropwise to the precursor solution keeping the same temperature and stirring for 5 min. Subsequently, sodium hydroxide ACS (NaOH) (CAS no. 1310-73-2) was added until the solution was brought to pH 10 and kept stirred for 20 min.

### Green synthesis of zinc oxide nanoparticles (ZnO NP)

The synthesis of ZnO NPs started with the zinc acetate ACS precursor (CAS No. 5970-45-6) at 0.21 M. The reaction was carried out on a hot plate until reaching a temperature of 70 °C with magnetic stirring. Then, 20 mL of aqueous extract of *C. sativum* was added with magnetic stirring for 4 h. Finally, the sample was calcined in a muffle for 2 h.

### Green synthesis of copper oxide nanoparticles (Cu_2_O NP)

250 mL of 0.05 mM copper sulfate pentahydrate solution was prepared and kept at 500 rpm using a magnetic stirrer. Next, 2.5 mL of 7.5 M sodium hydroxide (NaOH) was added dropwise. Finally, 10 mL of *M. dubia* extract and ascorbic acid were added dropwise, performing the in situ or post-synthesis impregnation of nanoparticles into the fabrics.

### Characterization of nanoparticles

The NPs were initially characterized by UV–vis spectrophotometry (UV 1900, Shimadzu) within the range from 350 to 900 nm. The size and shape of the NPs were analyzed by transmission electron microscopy (TEM), adding 5 µl of the colloid placed on a carbon-coated copper grid. The NP sample was then dried in a desiccator with silica for 16 h. Measurements were carried out on a JEOL (model JEM 2011) operated at a voltage acceleration of 120 kV. The crystalline structure was analyzed by X-ray diffraction (D8 ADVANCE model DAVINCI, MA, USA) with a Cu Kα source in the 2θ range of 20–80°.

### Bacterial and fungal challenges against fabrics with nanoparticles

*Pseudomonas aeruginosa* (ATCC No. 9027) and *C. albicans* (ATCC No. 10231) strains were grown in TSA medium and incubated for 24 h at 37 °C, and passaged and incubated for 24 h at 37 °C in TSB liquid medium.

Fabrics of 1 cm × 2 cm were challenged with Cu_2_O, ZnO, and Ag/Cu_2_O nanoparticles, placing the fabric segments in sterile 50 mL conical tubes with 1 mL of TSB solution of *P. aeruginosa* and *C. albicans* at a concentration of approximately 1 × 10^5^ CFU/mL and left to incubate for 24 h at 35 °C. Subsequently, 4 mL of new TSB solution was added, stirring for 1 min to detach the microorganisms from the fabric and inoculate in TSA by the streak seeding method in duplicate, leaving the plates to incubate for 24 h at 35 °C to perform the readings comparing the positive and negative control.

$$ \% {\text{ Bacterial reduction }} = { 1}00\left( {{\text{B}} - {\text{A}}} \right){\text{/B}} $$

where: A represents the Colony Forming Units (CFU) recovered from the nanoparticle challenge, and B represents the CFU recovered from the fabric without nanoparticles.

### Statistical analysis

A one-way Analysis of Variance was performed between the control and the treatments with a p < 0.05 of statistical significance. A Student's t-test was performed for dichotomous variables with p < 0.005 of significance. The free access statistical program Social Science Statistics was used https://www.socscistatistics.com/.

## Results

### Characterization of nanoparticles

**Figure 1:**
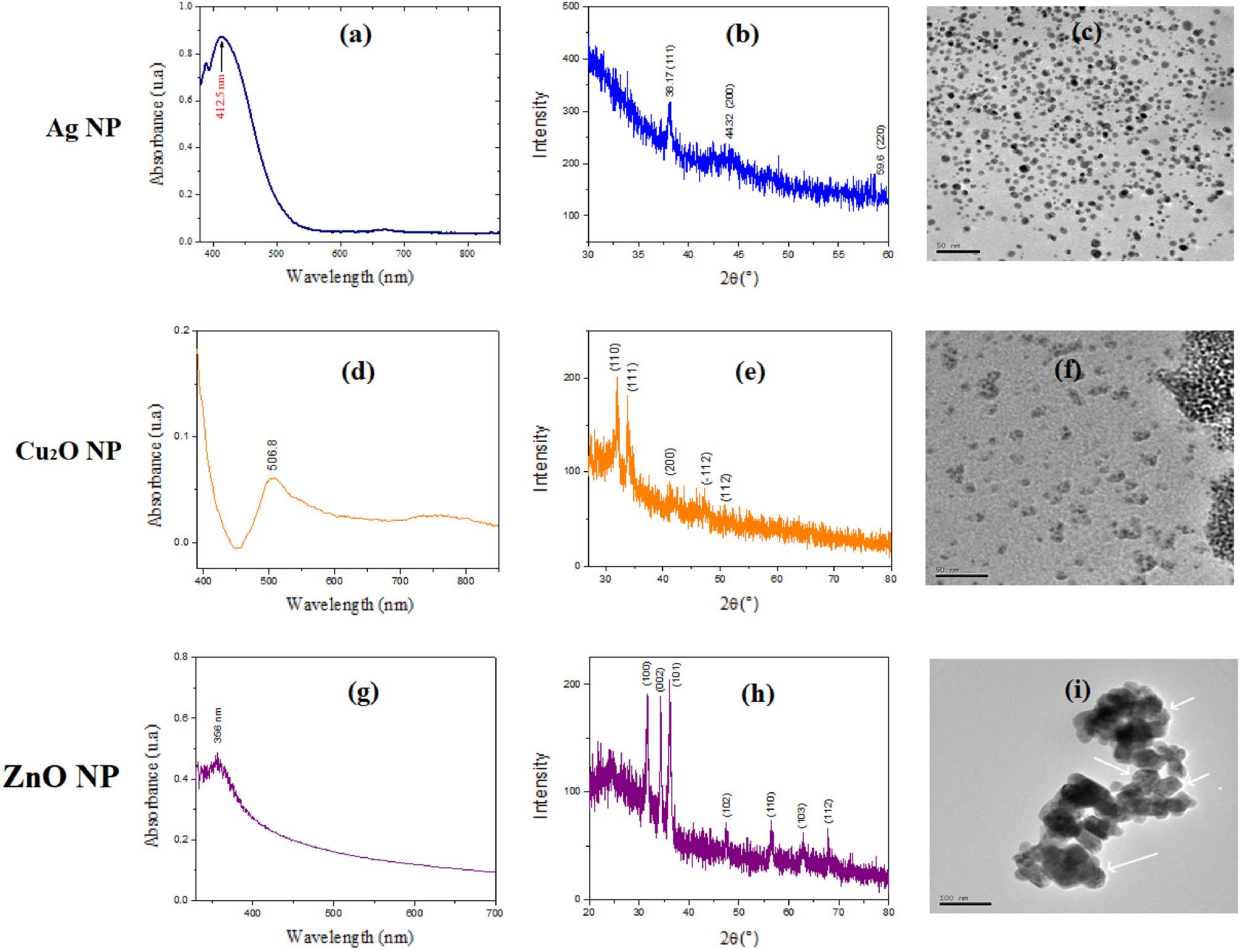
Characterization of Ag NPs (**a**) by UV vis spectrophotometry, (**b**) X-ray diffraction, (**c**) transmission electron microscopy (TEM). Cu_2_O NP (**d**) by UV vis spectrophotometry, (**e**) X-ray diffraction, (**f**) transmission electron microscopy (TEM). ZnO NP (**g**) by UV vis spectrophotometry, (**h**) X-ray diffraction, (**i**) transmission electron microscopy (TEM).

Figure 1a shows the spectrophotometric characterization of Ag NPs, a technique that confirms the formation and stability of this type of nanostructure, through the location of the plasmon peak. The characteristic band for this material can be evidenced by its peak located at 412.5 nm. The peak presented shows a blueshift, which implies the formation of smaller-size NPs as confirmed by TEM analysis^17^.

Figure 1b shows the X-ray diffraction (XRD) patterns for the colloidal Ag NPs. Two peaks can be observed at positions 38.1°and 44.3° corresponding to the (111) and (200) planes of silver as indicated by the JCPDS card, No. 04-0783. The results confirm that the Ag NPs are cubic crystals centered on the faces by the intensity of the (111) peak^18,19^.

The morphology and size of the Ag NPs were analyzed by TEM. The images shown in Fig. 1c indicate that the NPs are mostly spherical-shaped, with an average size of 6 nm. The NPs are shown dispersed with small clusters of various sizes^20,21^.

A broad spectrum of characterizations for Cu_2_O NPs is also shown. The formation of Cu_2_O NPs was initially confirmed by UV–vis spectrophotometry (Fig. 1d). An absorbance peak at 506.8 nm, characteristic of Cu_2_O NPs, can be evidenced^22,23^. The broadening of the peak indicates that the NPs are polydisperse.

The crystalline structure of the nanomaterial was also evaluated. In this sense, the X-ray diffraction patterns are shown in Fig. 1e, where the peaks show a high crystallinity with remarkable diffraction angles of 23°, 25°, 28°, 32°, 34°, 43°, and 47° corresponding to copper (Cu) with cubic structure centered on the faces^22,24^.

TEM analysis (Fig. 1f) gives us information on the shape, size, and distribution of the NPs. It was determined that the NPs have irregular shapes and, in some cases, almost-spherical shapes, with cluster formation. The synthesized Cu_2_O NPs have an average size of 10 nm, which is small compared to other publications^25^ and similar to others also synthesized by the green route^7^. It is suggested that the size reached is due to the influence of the capping effect of the extract.

For the ZnO NPs, we also started from the spectrophotometric characterization, attributing it to the exciton resonance. In this sense, the UV–vis absorption spectra of the ZnO NPs are shown in Fig. 1g, where the exciton peak at 355.5 nm corresponding to this nanomaterial is observed; however, a slight blue shift is observed, compared to that found by other authors^26–28^. Figure 1h evidences the X-ray diffraction patterns of the ZnO NPs. All diffraction peaks correspond to the characteristic hexagonal wurtzite structure and agree with the JCPDS card, No 36-1451^29,30^. The characteristic peaks correspond to lattice planes (100), (002), (101), (102), (110), (103), and (112). Figure 1i reveals irregular shapes with hexagonal and quasi-spherical tendencies with an average size of 50 nm joined in dispersed clusters, similar to those found by Al-Kordy et al.^31^.

The biogenic synthesis method appears to be a more sustainable, highly compatible, and low-cost alternative. On the latter characteristic, studies on this synthesis method carried out by one of the authors^32^ demonstrated the reduction of production costs by 84.24% regarding obtaining nanoparticle colloids from commercializing companies. The use of agro-industrial wastes, mostly for extracting bioactive compounds and, in turn, being applied as organic reductants, plays an important role in reducing costs.

### Antibacterial effect of nanoparticles

In the case of copper oxide, zinc oxide and silver/copper oxide nanoparticles, these showed 100% growth inhibition at the level of both *P. aeruginosa* and *C. albicans* by green synthesis and/or chemical synthesis (Tables 1, 2, 3).

**Table 1:** Effect of copper oxide nanoparticles (0.73 mg/mL) against *P. aeruginosa* and *C. albicans.*

	Fabric control (CFU/mL)	(Cu_2_O NP, in situ chemical)-(growth inhibition)	(Cu_2_O NP, in situ *M. dubia*)-(growth inhibition)	(Cu_2_O NP, post-synthesis *M. dubia*)-(growth inhibition)	(Cu_2_O NP post-synthesis chemical)-(growth inhibition)
*P. aeruginosa* (ATCC Nº 9027)	*1.15 × 10^5^ CFU/mL ± 0.06	*0.00 ± 0 (100%)	*0.00 ± 0 (100%)	*0.00 ± 0 (100%)	–
*C. albicans* (ATCC Nº 10231)	*1.05 × 10^5^ CFU/mL ± 0.06	*0.00 ± 0 (100%)	*0.00 ± 0 (100%)	*0.00 ± 0 (100%)	*0.00 ± 0 (100%)

*p < 0.001.

A one-way ANOVA was performed for independent data, resulting in a significant difference between the treatments and the control group.

**Table 2:** Effect of zinc oxide nanoparticles (0.099 mg/mL) against *P. aeruginosa* and *C. albicans*.

	Fabric control (CFU/mL)	(ZnO NP post-synthesis *C. sativum*)-(Growth inhibition)
*P. aeruginosa* (ATCC Nº 9027)	*1.15 × 10^5^ CFU/mL ± 0.06	*0.00 ± 0 (100%)
*C. albicans* (ATCC Nº 10231)	*1.05 × 10^5^ CFU/mL ± 0.06	*0.00 ± 0 (100%)

*p < 0.001.

A one-way Student's t-test was performed for independent data, resulting in a significant difference between the treatments and the control group.

**Table 3:** Effect of Ag/Cu_2_O nanoparticles (0.002–0.73 mg/mL) against *P. aeruginosa* and *C. albicans.*

	Fabric control (CFU/mL)	(Ag (*E. globulus*)/Cu_2_O (chemical), post-synthesis-(growth inhibition)	(Ag (*E. globulus*)/Cu_2_O (*M. dubia*) post-synthesis-(growth inhibition)
*P. aeruginosa* (ATCC Nº 9027)	*1.15 × 10^5^ CFU/mL ± 0.06	*0.00 ± 0 (100%)	*0.00 ± 0 (100%)
*C. albicans* (ATCC Nº 10231)	*1.05 × 10^5^ CFU/mL ± 0.06	*0.00 ± 0 (100%)	–

*p < 0.001.

A one-way ANOVA was performed for independent data showing a significant difference between the treatments and the control group in the case of *S. aeruginosa*. A one-way Student's t-test was performed for independent data, resulting in a significant difference between treatments and the control group for *C. albicans*.

## Discussion

**Figure 2:**
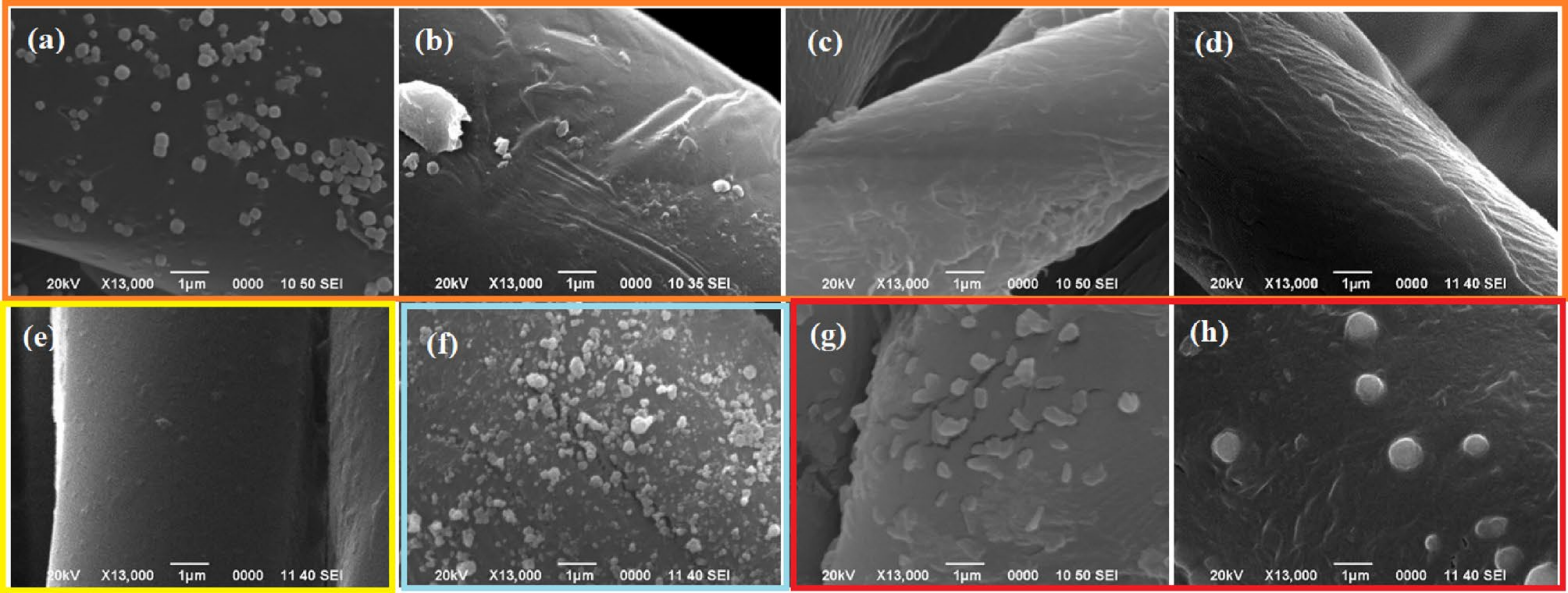
SEM images of textiles impregnated with nanoparticles. (**a**) Cu_2_O, in situ chemical; (**b**) Cu_2_O, post-synthesis chemical; (**c**) Cu_2_O, in situ *M. dubia*; (**d**) Cu_2_O, post-synthesis *M. dubia*; (**e**) Control textile; (**f**) ZnO, post-synthesis *C. sativum*; (**g**) Ag (*E. globulus*) /Cu_2_O (chemical), post-synthesis; (**h**) Ag (*E. globulus*)/Cu_2_O (*M. dubia*) post-synthesis.

The nanoparticles were impregnated in the textiles according to the following methodology: In the case of copper oxide nanoparticles obtained using the reductant ascorbic acid (A.A), two procedures were used: "in situ" textile treatment, i.e., the textile was submerged during the reduction process of the precursor, where it was ensured that the nanostructure formation phase would develop inside the textile fiber. Figure 2a shows the textile treated with the described protocol, where clusters can be seen on the fiber surface with defined morphologies. Similarly, Fig. 2b shows the fiber treated with Cu_2_O NPs obtained with chemical reductant but "post-synthesis," i.e., first, the NPs were synthesized, and then the textile was immersed in the solution where the nanoparticles are impregnated into the textile fiber. This can be evidenced in the SEM image by the formation of clusters without defining any specific geometry and of a larger size of NPs. This is evident since the impregnation of the colloid in the textile is not fully efficient and is mostly superficial. Something similar can be evidenced in Fig. 2c,d, which correspond to the Cu_2_O NPs obtained using the juice of *M. dubia* as a bioactive reducing compound treated "in situ" (during synthesis) and "post-synthesis" (after synthesis), respectively. In both cases, the presence of a coating can be observed, most likely linked to traces of organic material that did not react during the green synthesis process, showing in the first case a larger layer because the juice of *M. dubia* could not fully impregnate and enter the core of the textile fiber, not being able to fully react with the precursor. Figure 2e shows the control fiber without any treatment. Figure 2f shows the surface of the textile fiber treated with ZnO NP. As mentioned in the methodology, in this case, obtaining this type of nanostructure involves calcination processes, so the only way to perform the textile treatment is "post-synthesis." In the figure mentioned, clusters of ZnO nanomaterial can be evidenced in large quantities. This was also observable in other areas of the textile fiber previously visualized by SEM. Another type of treatment was through hybrid nanoparticles, i.e., by combining two types of nanostructures, as shown in Fig. 2g,h, showing textiles treated with Ag/Cu_2_O, using both organic extract (*E. globulus*) and chemical reductant (A.A), and in turn using organic media as the reductant in both cases, respectively. It is worth mentioning that, in these hybrid treatments, the nanoparticles were impregnated after synthesis (post-synthesis) in both cases, initially immersing the textile in the Ag colloid and then in the Cu_2_O NP colloid. In both cases, the presence of surface layers could be evidenced, possibly linked to an overaccumulation of silver nanoparticles that are smaller in size (6 nm), with the Cu_2_O NPs remaining in the outer layer mostly, and the presence of clusters corresponding to both types of nanomaterials could also be visualized.

It is noteworthy that the textile used for the nanomaterial treatment has 70% cotton and 30% polyester, with cotton guaranteeing a higher degree of impregnation.

*Pseudomonas aeruginosa* is an opportunistic pathogen causing a series of infections, especially of the upper respiratory tract, which results in an aggressive response from neutrophils causing tissue and systemic damage^17,18^. *Candida albicans* is an opportunistic pathogenic fungus that can generate biofilms and causes many infections called candidiasis in hospitalized patients with devices such as catheters or ventilation tubes, which may cause sepsis and hospital deaths^19,20^.

Previous studies have demonstrated the effect of copper and nanoparticles against bacteria, such as *Staphylococcus aureus* and *Escherichia coli*, notably reducing the bacterial load in 30 min at concentrations between 0.5 and 1 mg/mL, disrupting the reductase capacity of the bacteria and destroying the cell membrane^21–23^. In our study, the copper oxide nanoparticles against *S. aeruginosa* treated with the chemical In Situ method (A.A.) and both the green (*M. dubia*) In Situ and the Post Synthesis methods had a 100% growth inhibition (Table 1) of the bacteria, and the difference was significant compared to the control (p < 0.001).

A previous study of copper nanoparticles against *C. albicans* showed Minimum Inhibitory Concentration (MIC) ranges between 6.25 and 3.125 mM^10^. In our research, copper oxide nanoparticles at a concentration of 0.73 mg/mL showed 100% growth inhibition against *C. albicans* (Table 1) using both the chemical method and the green In Situ and Post-Synthesis method, being statistically significant when compared to the control (p < 0.001).

A previous study showed that zinc oxide nanoparticles at a concentration of 8 mg/mL prevented the growth of *P. aeruginosa*. The nanoparticles had a virulence-decreasing effect at 2 mg/mL concentration, affecting the Quorum System (QS)^24^. Another study has reported using nanoparticles against *P. aeruginosa* and the biofilm of this bacterium, showing that the minimum biocidal amount to kill 100% of the bacteria was 0.3 mg/mL and that the concentration of zinc oxide nanoparticles inhibiting biofilm at 90% was 0.03 mg/mL^25^. In our study, 100% inhibition of bacterial growth was at 0.099 mg/mL, obtaining an optimal antibacterial effect. Regarding the impact of zinc oxide nanoparticles at 0.099 mg/mL post-synthesis, they had a 100% inhibition effect on this fungus of public health importance involved in many candidemia (Table 2).

Previous studies of copper oxide nanoparticles on Gram+ bacteria such as *S. aureus* and Gram -, such as *E. coli*, have shown remarkable antibacterial effects^21–23^. Other studies of silver nanoparticles on *S. aeruginosa* have shown antibacterial effects related to the adherence of the nanoparticles at the membrane level, changing membrane permeability and internal cell turnover, especially proteins, and the generation of free radicals that cannot be handled by the bacteria leading to bacterial death with MICs between 0.001 and 0.2 mg/mL for strains and between 0.001 and 0.6 mg/mL for *S. aeruginosa* biofilm^7,26–28^. Copper oxide nanoparticles have also been reported to be effective against *C. albicans* at concentrations up to 3.13 mM^10^. Silver nanoparticles have been studied against *C. albicans* and the biofilm this fungus can form; *C. albicans* is an opportunistic agent in hospital infections and the oral cavity. Silver nanoparticles have been tested at 0.001 mg/mL with biofilm inhibitory effects^29,30^. In our study, a combination of plant and copper oxide at concentrations of (0.002–0.73 mg/mL), respectively, was used post-synthesis, with a combination of both a green/chemical and green/green reduction with 100% inhibition for *S. aeruginosa* and 100% for *C. albicans*, using the green/green post-synthesis method (Table 3).

Previous studies have highlighted using the green synthesis method as a safe, eco-friendly system, avoiding stable energy consumption and with optimal nanoparticle sizes^31,32^.

Finally, previous studies that have used silver nanoparticles (0.15 mg/mL for *P. aureginosa* and 0.06 mg/mL for *C. albicans*) and zinc oxide, although they have shown antimicrobial effects against *P. aureginosa* and *C. albicans*, have not have reached 100% inhibition of the growth of the microorganism^33,34^. In contrast, in our study, 100% inhibition of the growth of microorganisms was observed using the same type of nanoparticles.

Among the study limitations is that the assays are preclinical, and the cytotoxicity of the nanoparticles studied in human cells must be evaluated.

## Conclusions

It is concluded that copper oxide, zinc oxide, and hybrid (copper oxide/silver) nanoparticles synthesized by biogenic and chemical route had 100% inhibition of the growth of *S. aeruginosa* and *C. albicans*, they can be used as potential antimicrobials and highlighting their optimal antibacterial and antifungal effects.

## Data Availability

The original data are available upon request from the corresponding autor jesus.rojas.jaimes@gmail.com.
